# Case Report on Adrenal Schwannomas: A Rare Mimic in the Spectrum of Adrenal Masses

**DOI:** 10.7759/cureus.53619

**Published:** 2024-02-05

**Authors:** Pankhuri Garg, Jay D Dharamshi, Abhijit Dhale, Ruturaj Pendkar, Ghanshyam Hatwar

**Affiliations:** 1 General Surgery, Jawaharlal Nehru Medical College, Datta Meghe Institute of Higher Education & Research, Wardha, IND; 2 Urology, Jawaharlal Nehru Medical College, Datta Meghe Institute of Higher Education & Research, Wardha, IND

**Keywords:** multidisciplinary approach, histopathological examination, surgical exploration, diagnostic challenges, abdominal pain, adrenal schwannoma

## Abstract

Adrenal schwannomas are exceptionally rare tumors affecting about 0.2%, originating from the adrenal gland, presenting diagnostic challenges due to their nonspecific clinical features and overlapping radiological characteristics with other adrenal masses. Here, we report the case of a 49-year-old female with no significant medical history presenting with diffuse abdominal pain. Imaging studies, including contrast-enhanced computerized tomography (CECT), revealed a well-defined mass within the right adrenal gland. Given inconclusive radiological findings and persistent symptoms, surgical exploration was performed, leading to the identification and resection of the mass. Microscopic examination, including immunohistochemistry, confirmed the schwannomatous origin of the tumor. The final diagnosis of an adrenal schwannoma was established after a histopathological examination. Postoperatively, the patient was treated with antibiotics and discharged on oral antibiotics after suture removal on advised follow-up after 15 days. This case highlights the diagnostic complexities associated with adrenal schwannomas and emphasizes the necessity of surgical intervention for conclusive diagnosis. The report aims to contribute to the limited literature on adrenal schwannomas, enhancing our understanding of their clinical presentation and reinforcing the importance of a multidisciplinary approach in their diagnosis and management.

## Introduction

Adrenal schwannomas are sporadic tumors affecting about 0.2%, originating from the adrenal gland, and their occurrence within the medical literature is limited [1]. Schwannomas, typically associated with peripheral nerves, less than 0.2% of the 0.5-5% of all retroperitoneal schwannomas, seldom involve the adrenal gland, making them a unique subset of adrenal neoplasms [2,3]. The scarcity of documented cases contributes to the challenges in understanding the clinical presentation, diagnostic approach, and optimal management of these tumors. The adrenal gland is a common site for various neoplastic lesions, including adenomas, pheochromocytomas, and myelolipomas. These lesions often present with distinct clinical features and can be characterized through imaging studies. However, adrenal schwannomas present a diagnostic dilemma due to their nonspecific clinical manifestations, frequently overlapping with other adrenal masses [4]. The lack of pathognomonic symptoms poses a significant challenge for clinicians in distinguishing schwannomas from more common adrenal tumors.

Advanced imaging modalities, such as contrast-enhanced computerized tomography (CECT) and magnetic resonance imaging (MRI), play a pivotal role in characterizing adrenal masses. Nevertheless, the radiological features of adrenal schwannomas may not be distinctive and, in many instances, fail to provide a conclusive diagnosis, necessitating additional diagnostic measures [5]. Surgical exploration remains the gold standard for establishing a definitive diagnosis of adrenal schwannomas. Microscopic examination, including immunohistochemistry, is essential for confirming the schwannomatous origin of the mass and distinguishing it from other adrenal lesions [6]. Understanding the histopathological features of adrenal schwannomas is crucial for accurate diagnosis and subsequent patient management. Given the rarity of adrenal schwannomas, there is a paucity of comprehensive literature addressing their clinical characteristics, optimal diagnostic strategies, and long-term outcomes. Each documented case contributes valuable insights into the diagnostic and therapeutic challenges associated with these tumors [7].

This case report aims to enhance our understanding of adrenal schwannomas by presenting a detailed account of a patient with abdominal pain, highlighting the complexities in diagnosis and the significance of surgical exploration.

## Case presentation

A 49-year-old female with no significant past medical history presented to the Medicine Outpatient Department (OPD) of the super-specialty hospital of Wardha district, complaining of nonspecific abdominal pain for five hours. The pain was diffuse, without any specific exacerbating or relieving factors. The patient denied any associated symptoms, such as fever, weight loss, or changes in bowel habits. Physical examination revealed tenderness in the right upper quadrant of the abdomen, without any palpable masses or signs of peritonitis. Vital signs were within normal limits, and there were no apparent signs of hormonal excess, such as hypertension or palpitations. To investigate the cause of the abdominal pain, an abdominal ultrasound was performed, revealing the presence of a mass. Given these findings, further evaluation was deemed necessary.

**Figure 1:**
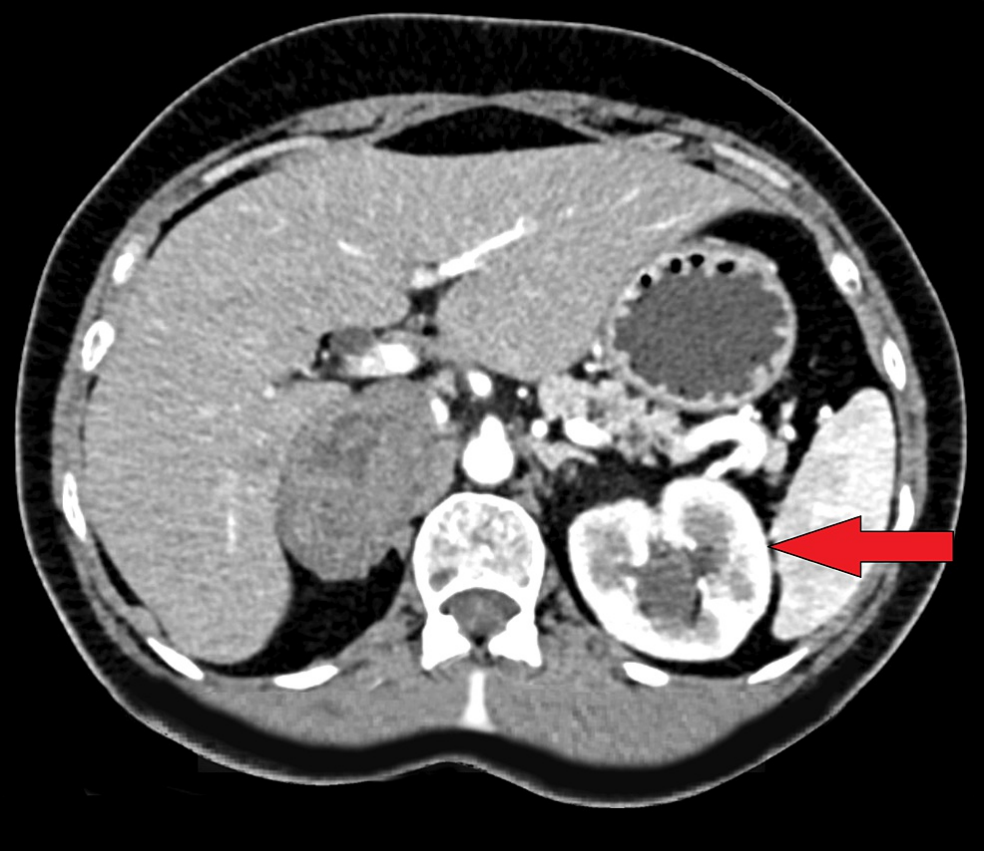
Contrast-enhanced computerized tomography (CECT) of the abdomen and pelvis shows a well-defined mass lesion within the right adrenal gland.

**Figure 2:**
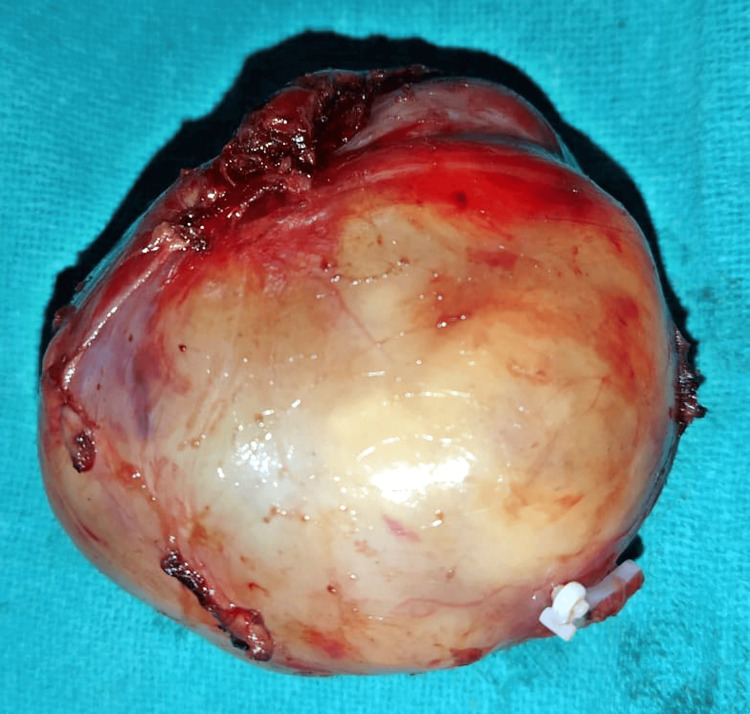
The largest lesion on the right measuring 8.5 x 7.2 x 6.8 cm and the left measuring 7.0 x 6.5 x 5.9 cm.

Contrast-enhanced computerized tomography (CECT) of the abdomen and pelvis revealed a well-defined mass lesion within the right adrenal gland (Figure 1). The mass exhibited an unusual oval shape with soft tissue attenuation, measuring 5.0 x 5.2 x 4.2 cm (Figure 2). Notably, the mean attenuation values were 23 Hounsfield units (HU) on the plain scan, 56 HU on the post-contrast phase, and 71 HU on the delayed 15-minute scan. In addition, tiny calcified foci were noted within the mass. The margins were smooth, and there was no apparent invasion into adjacent structures.

**Figure 3:**
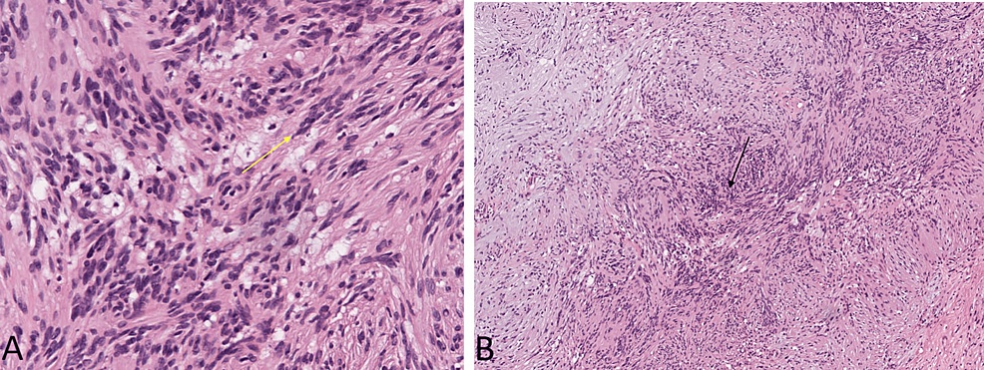
A) Nuclear palisading with tumor cells having an ill-defined cytoplasm with elongated tapered ends interspersed with collagen fibers (yellow arrow), 40x magnification. B) Biphasic population of tumor cells, 10x magnification).

Given the inconclusive nature of the radiological findings and the persistent abdominal pain, the decision was made to proceed with surgical exploration. Preoperatively, blood tests were performed, and an anesthetic call was done for fitness. Hence, after detailed counseling, written consent was obtained and the patient was shifted for a surgical procedure. Intraoperatively, a well-circumscribed mass was identified within the right adrenal gland. A surgical resection of the mass was performed. Microscopic examination of the resected mass revealed features consistent with schwannoma. Immunohistochemistry, including S-100 protein staining, confirmed the schwannomatous origin of the mass. The histopathological findings supported the diagnosis of an adrenal schwannoma (Figure 3).

The final diagnosis was established as an adrenal schwannoma, an exceptionally rare tumor originating from the adrenal gland. Adrenal schwannomas can masquerade as other adrenal masses, making their diagnosis challenging without histopathological examinations.

## Discussion

Adrenal schwannomas represent an exceedingly rare subset of adrenal tumors, with limited cases documented in the literature. The diagnostic challenges associated with these tumors stem from their nonspecific clinical presentation and imaging characteristics, often requiring surgical exploration for definitive diagnosis [8]. This case report contributes to the existing knowledge base by detailing the diagnostic journey of a patient presenting with abdominal pain ultimately attributed to an adrenal schwannoma.

The clinical presentation of adrenal schwannomas is nonspecific, and patients may exhibit symptoms such as abdominal pain, similar to other adrenal masses. This lack of distinctive clinical features underscores the importance of a comprehensive diagnostic approach, incorporating both clinical evaluation and advanced imaging techniques [9]. In our case, the patient's abdominal pain, coupled with tenderness in the right upper quadrant, prompted further investigation, leading to the identification of an adrenal mass.

Imaging studies, particularly CECT, are instrumental in characterizing adrenal masses. However, adrenal schwannomas can pose a diagnostic challenge as they may share radiological features with other adrenal tumors [10]. In our case, the CECT revealed a well-defined oval-shaped mass with soft tissue attenuation, underscoring the difficulty in distinguishing adrenal schwannomas from other adrenal lesions solely based on imaging characteristics.

Surgical exploration remains the gold standard for confirming the diagnosis of adrenal schwannomas. Microscopic examination, including immunohistochemistry, plays a pivotal role in differentiating schwannomas from other adrenal tumors. The biphasic population of tumor cells, nuclear palisading, and collagen fibers observed in our case are consistent with previous reports on adrenal schwannomas [11].

The differential diagnosis for adrenal masses is extensive and includes adrenal adenomas, pheochromocytomas, and myelolipomas, among others. Given the rarity of adrenal schwannomas, clinicians may not readily consider them in the initial differential diagnosis [12]. This case underscores the importance of maintaining a broad differential and highlights the necessity of surgical intervention for conclusive diagnosis in cases where imaging results are inconclusive.

## Conclusions

The presented case of an adrenal schwannoma illustrates the intricacies associated with diagnosing these exceptionally rare tumors. The patient's nonspecific abdominal pain and inconclusive radiological findings necessitated surgical exploration, leading to the identification and subsequent resection of the adrenal mass. Microscopic examination, including immunohistochemistry, played a pivotal role in confirming the schwannomatous origin of the tumor. This case emphasizes the diagnostic challenges posed by adrenal schwannomas, urging clinicians to consider them in the differential diagnosis of adrenal masses. The need for surgical intervention for a definitive diagnosis underscores the importance of a multidisciplinary approach, involving collaboration between clinicians, radiologists, and pathologists. With the limited literature available on adrenal schwannomas, this case report contributes valuable insights, highlighting the significance of continued research and documentation to advance our understanding of their clinical characteristics and optimal management.
